# Hospital and Institutional News

**Published:** 1913-08-23

**Authors:** 


### HOSPITAL AND INSTITUTIONAL NEWS.

### THE ROYAL PORTSMOUTH HOSPITAL.

In recent years we have frequently called attention to the condition of this institution and expressed a desire that steps should be taken to interest the people of Southsea in the work of the hospital, secure their co-operation, and in this and other ways make a determined effort to increase the ordinary income by £2,500 a year in accordance with the present needs of the institution. The present plan of living from hand to mouth with an annual ordinary expenditure which exceeds the ordinary income by upwards of £2,000 a year on an average is not satisfactory. It is not satisfactory either that the children's wards, which were built to accommodate twenty-six beds or cots, now contain each thirty patients, of whom no less than fourteen in one children's ward are women. On moral, hygienic, and other grounds, this condition of the wards is unsatisfactory, and the committee were wise in adjourning the consideration of the addition of five more beds to each children's ward by placing them in the balcony. We understand that, although the falling-off in annual subscriptions at present is small, the difficulty of obtaining new subscribers is great, whilst the working classes do not support this hospital as liberally as they did formerly. Directly the holiday season is over we hope that the committee will arouse themselves and encourage Mr. Wagstaff, the energetic secretary, to work actively a scheme whereby additional funds may be speedily forthcoming for the maintenance of this important hospital.

### BRENTFORD INFIRMARY'S MEDICAL SUPERINTENDENT.

Before the Brentford Union Board of Guardians rose for the vacation, a somewhat remarkable decision was arrived at, following upon the consideration of reports by Sir John Rose Bradford and Sir Thomas Barlow respecting the health of Dr. E. C. Austin, who was recently appointed to succeed Dr. Norton as medical superintendent of the infirmary. The appointment was made subject to a satisfactory medical certificate of Dr. Austin's physical fitness to undertake the work. Both examiners found Dr. Austin to have "a slight defect of the heart," but Sir John Rose Bradford declared that it was not "in any way serious or likely to incapacitate him." Sir Thomas Barlow expressed the same view even more emphatically, declaring that he considered him to be in sound health. In effect, Dr. Austin was said by two of the highest authorities to be medically fit for the position to which he had been appointed. Moreover, there was the additional evidence that Dr. Austin at Salford Union Infirmary has done exceptionally strenuous work for some years without the slight defect referred to developing into a serious one. It happened, however, that the advocates of another candidate formed a majority of the members present, and by ten votes to seven it was resolved to reject Dr. Austin and to advertise again. What the end of this will be no one can say. But should, as it is suggested, the British Medical Association decide to retaliate upon the Board for its treatment of Dr. Austin, the medical services of the Union may be seriously interrupted, and the sick poor will be the sufferers.

### THE LESSON OF THAW'S ESCAPE.

Mental hospital superintendents and prison authorities in this country must be congratulating themselves that their already heavy responsibilities have not included the supervision of Harry Thaw, who shot Mr. Stanford White, the New York architect, in 1906, and who has escaped from the Matteawan Mental Hospital in New York State. The Superintendent of this institution is Dr. Kieb, who is stated to have offered a reward of £100 for Thaw's capture, and to have suspended one of the attendants. The rumours that have been circulated in this country concerning Thaw's "romantic" escape certainly lend colour to the statements that collusion without and within the mental hospital is responsible for the success of Thaw's flight. Briefly, it appears that while the patients were exercising in the yard between breakfast and chapel a taxi-cab drew up at the entrance to the building, from which two prosperous-looking men got out. A few moments later one of the institution's employees came up to the gate, and as the door opened to admit him Thaw dodged the gatekeeper, rushed out, and jumped into the taxi. This immediately started down the road, where a high-powered motor-car was waiting, into which the fugitive and his friends scrambled and were soon able to out-distance all pursuit. Having passed beyond the boundaries of New York State, he possibly cannot be extradited, though it is ironically suggested that as an escaped "criminal lunatic" he might, according to the law of one State, be incarcerated anywhere as a menace to the public safety! What concerns us, however, is the probability of an elaborate and persistent intrigue, backed by limitless money, having infested the institution ever since Thaw's arrival there. The present is a very good example of the way in which the mere existence of a millionaire may become a public danger. For what institution or staff is likely to be able permanently to resist such corruption as Thaw's friends and relatives were certainly capable of exercising? The Superintendent, Dr. Kieb, has been placed in a position of extraordinary difficulty, and has had a most thankless task, one to which that of Sir Hudson Lowe at St. Helena seems to us no exaggerated parallel.

### THE ASYLUMS BOARD AND "SANATORIUM BENEFIT."

Beyond the mass of carefully correlated statistics and reports which form the bulk of the always instructive annual Reports of the Metropolitan Asylums Board, there are this year one or two features of novel interest. One is, of course, the account of the adaptation of the Downs School to the purposes of a sanatorium for tuberculous insured patients. The legal difficulties which had to be circumvented have already been fully dealt with in these columns, and it is sufficient to note that the circuitous route *viâ* the tripartite arrangement between the Insurance Committee, the County Council, and the Board led to the opening early this year of the sanatorium at Sutton for the reception of patients. Since then a portion of the Northern Hospital, Winchmore Hill, has been prepared for the accommodation of 200 patients. These two institutions together held at the beginning of June about two-thirds of the number of London insured persons receiving sanatorium treatment, an achievement which, having due regard to the difficulties and delays, must be considered creditable to the public spirit of the Board, though, in the light of more recent events, Mr. Burns' statement in the House of Commons to the effect that "not a single complaint has been made," and "no criticism can be wisely or fairly directed," has proved to be premature, at any rate as regards one of the institutions in question. The provision of further accommodation could no doubt be more easily undertaken by the Board than by any other authority, and at a vastly less capital expenditure, but such extension or readjustment could not be undertaken now, when, at a few months' notice, any arrangements made may be upset or cancelled.

### MEDICAL ASPECTS OF THE REPORT.

This fifteenth annual Report records a still further decline in the death-rates from scarlet fever and diphtheria, these rates being now respectively only 1.6 and 6.2, as compared with 2.5 and 8.8 in the previous quinquennial period. Of measles there were admitted even more cases than during the previous year, when a considerable epidemic was present in London. The death-rate (10.4) from this disease was also reduced as compared with last year, when it was 13.8. The death-rates of typhus and cerebro-spinal fever were 100 per cent.; but, as there were only two cases of each disease admitted, the figures are not so alarming as they seem. The year 1912 will be notable in the history of the ambulance service as the year in which horse traction was completely superseded by motor vans. The change has been very gradually made, and few of the old servants of the Board lost their positions on this account. It is satisfactory in these days to learn that, in spite of the large number of journeys made, no accident occurred involving injury to any passenger. Among the papers in the Medical Supplement note may be made of that by Dr. J. D. Rolleston on the treatment of diphtheria bacillus carriers with cultures of staphylococcus. This method, judging from the results in a very limited number of cases, appears promising. Mr. F. St. J. Steadman has presented a very practical contribution on the treatment of dental disease in children, which deserves attention. Tuberculin treatment is responsible, as might be expected, for several papers.

### IMPORTANT BEQUESTS TO HOMŒOPATHIC HOSPITALS.

The will of Mrs. Marion Pender Smart, of Tunbridge Wells, under which over £50,000 is left to charity, contains a bequest of £2,000 to the Tunbridge Wells Homœopathic Hospital, and a further gift of £10,000 to that institution on condition that a wing is built in memory of her husband and called "The Frank Smart Wing." She has left also £5,000 to the London Homœopathic Hospital. Her other hospital bequests include £1,000 each to the Convalescent Home, Beechfield, Worthing, the General Hospital, Tunbridge Wells, the Seaford Convalescent Home, the Home for Incurables, Putney, the Phillips Memorial Hospital, Bromley, the Dental Hospital, Leicester Square, the Hospital for Diseases of the Throat, Golden Square, W., and St. Mark's Hospital, City Road; also £500 to the Eye and Ear Hospital, Tunbridge Wells. The gross value of Mrs. Smart's estate is given as nearly £700,000.

### LADY HAMBLEDEN AND KING'S COLLEGE HOSPITAL.

Among the few honoured names of those who have been created peeresses in their own right is that of Viscountess Hambleden, who died last week at the age of eighty-five. Her life in its devotion to philanthropic activities, more especially in connection with King's College Hospital, bears a resemblance to that of the late Lady Burdett-Coutts, who also, of course, was created a peeress by Queen Victoria for her philanthropic enterprise. Born in 1828, the daughter of Mr. F. D. Danvers, she first married in 1854, and after becoming a widow within twelve months, Mrs. Leach, to give her married name, was wedded a second time four years later to Mr. W. H. Smith, afterwards leader of the House of Commons, and then virtually the head of the well-known firm. On his death in 1891 Queen Victoria offered her a Viscountcy as a mark of the Queen's sense of Mr. Smith's public work, notably in connection with King's College Hospital, a work in which Lady Hambleden had always taken an active as well as a most sympathetic interest. This work she continued unabated, giving indeed no fewer than seventeen different donations to the removal fund. As the Viscountcy carried with it remainder to her heirs male, the title devolves on the Hon. W. F. D. Smith, the well-known chairman of King's College Hospital, on whose work a special illustrated article appeared in The Hospital of March 11, 1911.

### THE LONDON SCHOOL OF TROPICAL MEDICINE.

Owing to the generous response which was made to Mr. Austen Chamberlain's appeal on behalf of this School new buildings are being erected in connection with it at the Albert Dock, which have made it one of the most complete schools of medicine in the kingdom. We propose to give a full account of the School as it is to-day in our Educational Number, which will be published on September 6.

On the 8th instant, by the invitation of the School Committee, the Tropical Section of the International Medical Congress visited the London School of Tropical Medicine. There was a large attendance of distinguished professors and physicians from practically every country in the world. The guests were received by Dr. F. M. Sandwith and Dr. G. C. Low, and introduced to all the physicians and teachers in the School. Amongst those present were Sir Patrick Manson, Dr. James Cantlie, and Professors Blanchard, Laveran, and Nuttall, with Mr. P. J. Michelli, the able secretary, whose energy and devotion have contributed greatly to the high efficiency of the present organisation and buildings. Great satisfaction was expressed, and the visit cannot fail to have an important bearing upon the influence and work of an institution which must increasingly add to the safety and welfare of the race throughout the British Empire.

### DEATH OF A WELL-KNOWN MEDICAL MAYOR.

The death, on August 15, of Mr. Frederick Montague Miller, M.R.C.S., L.R.C.P., formerly of Upper Clapton, removes one who, until recently, was quite one of the best-known medical men, and most marked personalities in the North-East of London. Mr. Miller, who was sixty-five at the time of his death, had practised for many years in the High Road, Upper Clapton, and had also taken an active part in local politics. He had twice been Mayor of Hackney, and was a Justice of the Peace. Of enormous frame and proportions, Mr. Miller was one of those whose very appearance suggests geniality mingled with robust commonsense; and his appearance did not belie him, for those were his leading attributes. He possessed large private means, which would have tempted most men into laziness; but he worked on just the same in Clapton, where hundreds of poor people remember him with affectionate regret. Some few years ago the police, quite mistakenly, brought against him a charge of cruelty to a horse; this he successfully refuted, and, indeed, no one who knew him could believe it to be true. Mr. Miller was educated at St. Thomas's Hospital, whence he qualified in 1869.

### LOCAL GOVERNMENT BOARD AND THE CONTROL OF SYPHILIS.

The general tenor of our leading article last week on the coming control of syphilis has been confirmed to a remarkable extent by the Report just issued by the Local Government Board. This Report has been drawn up by Dr. R. W. Johnstone, one of the medical inspectors of the Board, and contains, besides interesting matter on the history and incidence of syphilis, the results of his inquiry into the control over venereal diseases, with special reference to the adequacy and general character of the arrangements for institutional treatment of these diseases now available in England and Wales. As confirming the opinion expressed in our last issue, we cannot imagine anything more apposite than the following paragraph which we make no apology for quoting in full:—"It cannot be too strongly urged," says Dr. Johnstone, "that the best method of controlling venereal diseases and protecting the free from infection would be the provision of means for early and accurate diagnosis, with skilled advice and adequate treatment available for all infected persons. In short, the essence of the problem is how to get a willing patient at the earliest time to the doctor from whom, or to the institution from which, such advice and treatment are to be had." How best to secure the means for carrying out the much needed adequate treatment will doubtless form the subject of further inquiry. It is probable that the provision of accommodation in existing general hospitals would be the best plan to adopt rather than the erection of special hospitals for this class of disease. This view is the one taken by Dr. Arthur Newsholme in his Introduction to this Report, to which the attention of all institutional workers may be profitably directed.

### THE FALLACIES OF THE RETRIBUTIONISTS.

The Report is in emphatic agreement with our contention that "regulation" as a method of dealing with venereal disease is a complete failure. Seeing that males form the large majority of infected and infectious individuals it is unscientific and unprofitable, as well as manifestly unjust, to apply repressive legislation directed against a particular class and sex of sufferers who are neither the most dangerous nor the most numerous. It is easily comprehended that such action cannot fail to assure the resentment and active opposition of those capable of considering the problem judicially. Dr. Johnstone agrees that the notification of venereal diseases is at present impracticable and premature. It is certain that any attempt at notification would be futile for practical purposes and would greatly increase the number of concealments. Such concealment inevitably means that resort to quacks and self-treatment would become much more common even than at present, and the results to the community would be disastrous. Even the impersonal notification employed in Denmark has proved of little value and is decidedly misleading in the matter of statistics owing to overlapping. Until adequate provision for efficient treatment, now for the first time scientifically feasible, is within the reach of every sufferer without any deterrent stigma attached, full notification may be left out of consideration. What is needed is that a more reasonable attitude towards the whole subject should penetrate the public mind and that the point of view of the retributionist be regarded as obsolete and calamitous. Such books as "Prostitué" by Victor Marguerite have done something to educate the public, but the above conception of venereal disease has proved, and will prove, a great difficulty and hindrance in the battle against these destructive plagues.

### TUBERCULOSIS APPOINTMENTS.

More than once attention has been drawn in these columns to the haphazard, and often highly unsatisfactory, way in which local bodies have been allotting appointments as tuberculosis officer.

A better example of the way not to set about this business could hardly be given than one explained in the correspondence columns of last week's *Lancet*. A medical man who has specialised for nine years in sanatorium work applied for a chief tuberculosis officership in an adjoining county, for which post he was very highly recommended by the medical officer of health of his own county. He heard nothing as to the fate of his application (not even an acknowledgment of it, though he found out that it had not miscarried) until he got a note from a young doctor asking permission to call to get a few hints, as the latter had just got the post! On receiving his visitor, the sanatorium superintendent found out that the newly appointed chief tuberculosis officer had no tuberculosis experience and no diploma of public health; could not read German and was quite unfamiliar with English tuberculosis literature; had never seen the artificial pneumothorax treatment; and knew nothing of laryngoscopy! This seems a distinct case for the interference of the Local Government Board.

### THE STAINING OF LIVING TISSUES.

THE death last week of Professor Edwin Goldmann, of Friedburg University, removed one of the foremost workers in cancer research. Much of his active life was spent at Freiburg, where he held many posts, including those of assistant at University Hospital, Professor of Surgery at the University, and later senior surgeon to the Deaconess House. His great work was, of course, on the staining of living tissues and the nature and importance of cell division in the spread of cancer. His publications on malignant growths and on "external and internal secretions in the light of vital staining," together with a contribution upon "the application of $x$-rays for the diagnosis of cancer" indicate something of the immense industry with which he pursued his microscopic investigations. He was only fifty years of age, and, by a coincidence well known among scientific research workers, fell a victim to a form of the disease which during his whole life he had been busy in investigating. Professor Ehrlich has written to the British press urging that Goldmann's work and research should be carried on by the endowment of an institution to his memory. He (Ehrlich) is convinced that the special line of research with which the deceased Professor's name was associated is one of the greatest importance in the study of cancer problems. As a matter of interest we may note that Professor Goldmann was a British subject.

### FROM MENTAL HOSPITAL TO MEDICAL SCHOOL.

THE visit of the Psychiatric Section of the Medical Congress to Cardiff Mental Hospital last week was noteworthy in several respects. In proposing the health of the guests at the Guildhall luncheon, Alderman Thomas Morgan, the Lord Mayor, reminded his hearers that the President of the Psychiatric Section, Sir James Crichton-Browne, was the former medical superintendent of the Wakefield Mental Hospital, where the first research laboratory for mental disorders was established. The research into the causation of insanity which had begun at Wakefield was now continued at Cardiff and several other institutions; but, as Parliament had voted grants for the eradication of tuberculosis, he hoped that the Home Secretary would soon help them in this direction. Sir James Crichton-Browne, in reply, said that mental hospitals would more and more become medical schools where medical students would receive instruction enabling them to recognise and treat baffling forms of incipient nervous disorders. The work of the Cardiff Mental Hospital Committee had been notable, for they had erected buildings thoroughly well equipped and well planned, with all the modern hygienic improvements and appliances. It had a committee of visitors, who had adopted enlightened views, and he could not refrain from saying publicly that Dr. Goodall was one of the most distinguished of the medical psychologists in the United Kingdom. Dr. Goodall's achievement in acquiring a thorough knowledge of the Welsh language within twelve months of his arrival in Wales was also alluded to. The value of the meeting, however, was seen in the help it will give to the public recognition of the importance of mental research, and to the need for its adequate endowment and organisation.

### A GUARDIAN'S HOBBY.

AT an inquest last week concerning a death in the mental ward of St. Pancras Workhouse, which was held at the instance of the deceased's widow, who expressed herself unsatisfied at the treatment accorded to her husband, more than one point of interest arose. Dr. Arthur Chilcott, medical superintendent of the St. Pancras Infirmary, and Dr. Christopher Thackeray, gave evidence, and the jury, in returning a verdict of "death from natural causes," added a rider that in their opinion the patient was properly treated. The Coroner concurred, as the evidence foreshadowed that he would. In addition, however, to this expert testimony—and, by the way, an independent *post-mortem* examination was held at the Coroner's instance, so the widow, we hope, will be satisfied of her mistake—Mr. W. R. Curtis, of Regent's Park Road, also gave evidence. As a member of the Board of Guardians and vice-chairman of the visiting committee, he stated that his hobby for some years had been to visit every Sunday the ward in which the deceased had been accommodated. This statement bore out what one of the infirmary sisters had said earlier in the inquiry, and there seems to be no doubt that any complaints that were felt in this ward would be confided to Mr. Curtis, and so find their way to the Guardians' ears. Not very long ago we had to record a case of injudicious visiting on the part of a provincial Guardian. We are glad now to have so much more satisfactory an instance to quote. When visiting becomes a hobby it rises on to a different plane, a plane where, with due discretion and experience, it may be conducive to much good.

### THE WARD POISONS CUPBOARD.

At a recent inquest on a two-year old child which had died in the Wolverhampton General Hospital, it was stated that the child had been found with a poison bottle in its hand. It appears that it was the custom to keep one or two bottles for emergency use, which contained poisons, on a table in the children's ward, and it is thought that in the process of cleaning the ward the cot containing the child was moved, thus enabling it to obtain the bottle, which apparently contained strychnine. The jury returned a verdict of accidental death, adding a rider that poisons should be kept locked up—a rider with which we heartily concur. To the hospital visitor who keeps his eyes open, it is no unknown sight to find inside, or just outside, the wards the poison cupboard with the door conveniently ajar. We have also often observed the cupboard locked, but not infrequently with the key in the door, which can hardly be considered an improvement.

### EFFECTS OF OPEN-AIR TREATMENT ON ARCHITECTURE.

In a recent article on the great public children's hospital—Queen Mary's Hospital for Children at Carshalton—attention was directed in The Hospital (July 26) to the separate cottage blocks with their verandahs, which, forming three sides of a quadrangle, enabled large numbers of the children to be treated in the open air. A somewhat similar type of building is being designed by Mr. John Stuart, architect to the West Riding County Council, for an open-air school at Highfields, a Yorkshire mining village. While keeping the quadrangular form, the new school is modified in such a way as to admit both sides of the class-rooms to be thrown open, thereby allowing the children to study in a building more resembling a wall-less shelter than a room. The rooms are furnished with French windows, and even the corridor leading to the rooms is provided with sliding windows, which cease about eighteen inches above the floor. In the article above referred to, Dr. Gordon Pugh, the medical superintendent of Queen Mary's Hospital, expressed the view that the cottage block was probably the form of hospital architecture of the future, and that more and more hospital patients generally would be treated in the open air. We recently commented on the growth of the country hospital, and the passing of the closed ward. At the present time of transition, for the last word in hospital architecture has certainly not been said, it is instructive to note every architectural step towards open-air buildings.

### SEPTICÆMIA AND MISSION HOSPITALS.

The death of Mr. A. H. Browne, stationed at Amritsar, in the Punjab, from septicæmia, adds a fourth to the list of those medical missionaries of the Church Missionary Society whose death has occurred from this cause during the past fifteen months. He was a Glasgow man, and graduated M.B., C.M., in 1890, taking honours in surgery, being John Hunter medallist, besides being also a prizeman in gynæcological medicine. Six years after taking his degree he left for India, and, maintaining his already keen and trained interest in scientific studies, produced a monograph on "Bubonic Plague," which was specially designed to aid his fellow missionaries. It is suspected that Mr. Browne contracted septicæmia from one of his patients in the Amritsar Hospital.

### THE WELSH SANATORIA COMPETITION.

It will be remembered that last year the Welsh National Memorial Association invited competitive designs from architects for new sanatoria, and after a considerable amount of bungling, by which competitors were the chief losers, the whole competition was indefinitely postponed. The circumstances were reviewed in our issue of July 6, 1912, and, after a year of silence, architects gave fresh vent to their grievance in the professional papers. It is, therefore, interesting to learn that a fresh competition has been organised, an independent assessor appointed to adjudicate, and it is gratifying to know this new competition is restricted to those who entered for the original competition. We congratulate the promoters on this solution of the difficulty and hope they will obtain an excellent design. The site is a good one, with a fall of some 70 feet towards the south, and is situated four miles from Denbigh. The conditions ask for accommodation for 184 patients, with provision for a further 100 in the future. Designs are to be submitted before September 30.

### THE MULTIPLYING OF COUNTY SANATORIA.

It is announced that the Kent County Council have decided to build a central sanatorium for Kent, provision being made for 100 beds, and the expense on the rates to be £6,000. Now, as the Local Government Board provide three-fifths of capital expenditure to two-fifths furnished by local bodies, it would seem that this institution is to be erected for £15,000, or at the statutory rate of £150 a bed. It will be interesting to see if this really can be done in practice. The question of site will become important, for when cost is limited, proximity to water and sewage mains, the former at any rate, would be a great help. £150 per bed does not leave much over for a steam laundry either, and probably the washing will have to be "put out," sometimes a difficult matter where consumptives are concerned. In Cornwall, not so rich a county as Kent, the County Council, through their finance committee chairman, Mr. R. G. Rows, announce their intention of purchasing an existing hotel, to house sixty patients, for £7,500; thus at perhaps about the same rate, or not much less, for a fair amount of alteration will have to be undertaken. All such efforts at economy are to be encouraged, in view of the widespread anti-tuberculosis efforts now in progress, and we hope to see similarly reasonable views prevailing in the matter of staffing. One thing is certain; and that is that the country cannot afford to maintain all these new institutions if run on current general hospital lines. The model should rather be those of sundry old-established private sanatoria, which, too, have accommodated patients decidedly superior in social class to beneficiaries under the National Insurance Act. If this point be not kept well in view, then in about two years' time, when the pinch comes, with dozens of these institutions to be kept up, there will be in all probability a considerable outcry. We repeat, with the distribution of wealth as it is at the present time, the care of consumptives must be conducted with the greatest regard for economy.

### A COLONY FOR THE FEEBLE-MINDED ON TYNESIDE.

Subject to the sanction of the Local Government Board, an important scheme for providing for the feeble-minded and epileptic is to be carried out near Newcastle. The public bodies responsible for it are the Poor-Law Unions of Northumberland, Durham, Westmorland, and Cumberland, which have formed a provisional joint committee, and it has been decided to purchase a house with 140 acres of land for adaptation into a colony at a cost of £13,000. As the building of the hall is said to have cost nearly three times this sum, the joint committee are being congratulated upon their bargain. The cost of adaptation and furnishing is estimated to be a further £17,000. The Chairman of the joint committee, which has succeeded in persuading so many different Unions to combine for this purpose, is the Rev. Samuel Pearson, of Tynemouth, and the Secretary is Mr. J. W. Coulson, clerk to the South Shields Board of Guardians. Their decision, coinciding as it does with the passing of the Mental Deficiency Act, should entitle them to a grant, and the scheme should further be an improvement upon the prevailing system under which the different Unions maintain their mentally deficient and epileptic cases, sometimes through out-relief, sometimes in workhouses, and sometimes in special institutions. The new scheme should guarantee the special provision and particular training which have now proved their value.

### X-RAYS AND THE SMALLER HOSPITALS.

An important meeting was held last week at the Albert Infirmary, Winsford, an institution with sixteen beds, to consider a report from the secretary, Mr. J. H. Cooke, on the proposed installation of *x*-ray apparatus. Mr. W. H. Verdin presided, and estimated the cost of the scheme at £600, but he thought there should be some indication of how much support was coming before the installation was actually decided on. He proposed to give £50 and the land required for the building. In reply to Dr. Okell, Mr. Cooke instanced Malton in Yorkshire and Alnwick as towns of a similar size to Winsford at which the infirmaries were fitted up with *x*-rays. The board decided that the installation of the *x*-rays and electric lighting was desirable and authorised the secretary to receive subscriptions. The report and the meeting have a twofold interest. In the first place it is very encouraging to see a small institution, like the Winsford Infirmary, thoroughly alive to the importance of being up to date and fully equipped for its work, and Mr. Cooke is certainly to be congratulated for the great trouble, which, as is evident from his report, he has taken to find out from Mr. Woods, who has charge of the *x*-ray department at the Liverpool Royal Infirmary, the expense likely to be involved, to compare tenders, and examine at the same time the cost of installing electric light. There are many small hospitals which are unprovided with electric light or *x*-ray apparatus, and the above is a good example of the way to set about acquiring them for which they may well be grateful.

### "THE HOSPITAL'S" SPECIAL EDUCATIONAL NUMBER.

On September 6 we propose to publish a Special Educational Number of The Hospital in accordance with our usual custom at this time of year. It is sometimes rashly supposed that institutional officers are not directly concerned in the considerations which weigh with both medical students and the newly qualified men. No mistake could be greater. In an article which we hope to publish in the Special Educational Number dealing with "The Scarcity of Resident Medical Officers" we shall show how intimately are the interests of hospital administrators bound up with the medical schools and with the ideas or fashions which rule there. Nor can the position with regard to hospitals and resident medical officers be understood by the administrator until the position and ideas of the medical student and the newly qualified are clearly grasped. The medical student and the man fresh from the schools cannot make a wise choice, moreover, until the position of the hospitals *qua* appointments is explained to them. A great change, too, has come over the profession of medicine during recent years, and particularly during the last two or three. The opportunities offered in the many new Services have multiplied to a great extent. We, therefore, shall treat in the Special Educational Number of the new professions now open to medical men. Last year, in addition to information concerning the Army and Navy Services, the new school appointments and the prospects attaching to them were dealt with. This year a much wider area will be covered, and the other two great spheres of work which have been set on foot in the intervening period will be exhaustively dealt with. Further particulars will be announced next week. The price of the Special Number will be, as usual, one penny.

### THIS WEEK'S DRUG MARKET.

Few price changes of importance have taken place during the week and, as is usually the case in August, business in drugs is quiet. A fair amount of quinine has changed hands, buyers being anxious to cover their requirements before a further advance in prices takes place. The high price of citric acid is well maintained, and any change that may take place is more likely to be in an upward than in a lower direction. Cod-liver oil is a very quiet market and quotations are practically unchanged. Cream of tartar is tending dearer. The price of essence of lemon is on the down grade. Quotations for strychnine have been reduced. The Opium market continues quiet.

